# Case of Alopecia

**Published:** 1878-12

**Authors:** S. W. Gillespie

**Affiliations:** Sterling, Ill.


*A case of Alopecia.*

During my attendance on a family living near this city, my attention was called to one of the children, who had for years suffered from alopecia, and as it presented some interesting peculiarities, I have thought it worth while to make note of it, with the hope that some of the readers of the JOURNAL AND EXAMINER may throw some light on its cause.

The patient, if I may call her such, is a very bright girl of eleven years of age, exhibiting in all respects the intelligence and characteristics of any healthy child of that age, and was, as her mother informed me, very forward in her studies at school. When between four and five years old her hair, which was very thick, long and curly, and of a dark brown color, commenced to come out in patches all over her head, leaving the head perfectly bald in these places, which varied in size from a kernel of coffee to that of one of the old fashioned pennies. In a little while the hair would be replaced in these spots and her head would look as well as before. This alternate falling out and replacing of the hair occurred, the mother says, two or three times, and finally was followed by the loss of all the hair, which came out gradually, leaving the head perfectly smooth and bald.

At the present time the child's head resembles very much that of a man of seventy years, who became bald before his hair turned gray. Over the ears, and extending from ear to ear around the base of the head there is a slight growth of silvery white hair, varying in length from half an inch to one inch and a half. The crown of the head is perfectly smooth and bald, the hair follicles looking very much as they do when they have become atrophied from old age. At the point where we would look for the posterior fontanelle there exists an oval depression into which the end of the first finger can be placed. There is, however, a perfect floor covering the membranes of the brain at the bottom of this opening, and it seems as if only the inner table of the skull had united, leaving the outer table open. Otherwise the head is perfectly normal. The most singular thing about the case is that there was no apparent cause for the loss of hair. The mother has no recollection of the child's having any fever or other disease previous to or during the time it suffered in this way, and says that she has always been very healthy.

The parents are both healthy, and present no symptoms of specific disease. They have seven other children, all of whom are healthy in every respect, and they can give no information which will explain the cause of this singular attack of alopecia.

S. W. GILLESPIE, M.D.

STERLING, ILL.

[This is evidently a case of alopecia areata, or area Celsi, due to perverted innervation, most often encountered in children, the treatment for which is described in every good treatise on skin diseases.—ED.]

